# DERMA: A Melanoma Diagnosis Platform Based on Collaborative Multilabel Analog Reasoning

**DOI:** 10.1155/2014/351518

**Published:** 2014-01-21

**Authors:** Ruben Nicolas, Albert Fornells, Elisabet Golobardes, Guiomar Corral, Susana Puig, Josep Malvehy

**Affiliations:** ^1^La Salle, Ramon Llull University, Quatre Camins 2, 08022 Barcelona, Spain; ^2^Melanoma Unit, Dermatology Department, Clinic Hospital, Rosselló 149, 08036 Barcelona, Spain

## Abstract

The number of melanoma cancer-related death has increased over the last few years due to the new solar habits. Early diagnosis has become the best prevention method. This work presents a melanoma diagnosis architecture based on the collaboration of several multilabel case-based reasoning subsystems called DERMA. The system has to face up several challenges that include data characterization, pattern matching, reliable diagnosis, and self-explanation capabilities. Experiments using subsystems specialized in confocal and dermoscopy images have provided promising results for helping experts to assess melanoma diagnosis.

## 1. Introduction

Melanoma is growing in importance because it is increasingly more prevalent in our society and it affects people of any age. Although it is not the most common skin cancer, if it is not early treated, its mortality is around twenty percent, according to the American Academy of Dermatology [1]. The most important difficulties related to early diagnosis are that it is a problem with a non trivial classification process, given the high volume of data, different experts, and types of diagnosis and the fact that there are not enough clear classification patterns. The characteristics of the problem have fomented the application of artificial intelligence techniques to exploit data and help experts in the early diagnosis. This work describes DERMA, a melanoma diagnosis architecture created as a result of the collaboration between the department of dermatology at the Hospital Clinic of Barcelona (HCPB) and the Institute of Biomedical Research August Pi i Sunyer (IDIBAPS). DERMA is a collaborative architecture among several subsystems specialized in different kinds of data sources. More specifically, the current version is based on the collaboration of two multilabel case-based reasoning (CBR) [2] systems that use confocal and dermoscopy images, respectively, which are the most important image analysis in melanoma cancer to date [3, 4]. CBR is used as an engine due to its self-explanation capabilities extracted from solving new problems from past experiences, which are important for experts to understand the results. Thus, on the other hand, the multilabel mode [5] means that a new case is classified in several subclasses, so this additional information helps experts to understand the melanoma case more accurately because complex patterns are better described as a set of simpler patterns. The collaboration of CBR systems enables the replication of the medical protocols from the different expert profiles. Moreover, the CBR data is reorganized using distance metric learning [6] to promote the separation between malignant and nonmalignant cases. Therefore, distance metric learning and the multilabel collaboration scheme result in an improvement in the sensitivity and specificity of the diagnosis, which is precisely what medical experts are looking for.

The following sections are described as follows.  Section 2 summarizes some related work to contextualize the proposal.  Section 3 presents the DERMA architecture and each one of its modules. Next, Section 4 highlights the main results obtained. Finally, Section 5 ends with the conclusions and further work.

## 2. Related work

Artificial intelligence (AI) techniques have been used with outstanding results in different knowledge areas such as marketing, education, or medicine. This is because these kinds of problems have so much information which is not directly processable by the human mind. To overcome this difficulty we need techniques to extract patterns and to deal with this information. Nowadays research in artificial intelligence for cancer is an outstanding research topic supported by different institutions and contests such as the Google Science Fair which awarded a breast cancer research project in 2012 or the Intel International Science and Engineering Fair 2013 that won a leukemia project.

The range of artificial intelligence techniques applied in medical problems is large and covers four groups of data mining methods evolved in these processes: clustering, association rules, classification, and regression. Regardless of the outstanding groups which are clustering, the goal is to decompose the problem and try to model it in a proper manner. Therefore, association rules, which focus the interest on why things happen, are of great relevance. In some cases, clustering and association rules are used as base methods to apply classification and regression processes. Considering these characteristics we could break the problem into two frequent groups: methods to find patterns and tools to aid decision making (recommender systems) with some examples of works on these areas.

The objective of aiding decision making has been addressed in several types of cancer such as breast cancer and has been treated from different points of view [7]. One goal is to achieve an automatic feature extraction from breast images in order to detail their characteristics. The work performed by [8] meets this objective through the use of *k*-means and fuzzy *c*-means clustering techniques. With the aim of avoiding invasive techniques with psychological, health, and economical consequences, some works that use techniques such as thermography for diagnosis appear. An approach using Bayesian networks is presented by [9] in order to fit this target. DESMAI framework [10] allows experts in breast cancer to explore digital mammography databases according to a certain topology criteria when they need to decide whether a sample is benign or malignant. This work was performed through a variant of a case-based reasoning system featured by organizing the case memory using self-organizing maps (SOM). In melanoma cancer, one approach is to construct a domain model that combines learning methods with different characteristics [11].

Considering the recommendation methods group we found [12] which proposes an automatic way to build decision support systems by means of combining several machine learning techniques using a metalearning approach based on grammar evolution. In the particular case of melanoma cancer there are works that enable knowledge discovery such as [13] that uses SOM to identify groups of similar melanoma and creates descriptions of clusters that are used as explanations for experts. To support dermatologists in assessing the classification of skin lesions using dermoscopy in order to asses prior to extraction, [14] uses a combination of case-based reasoning and clustering for generating a domain theory to classify melanomas in situ. In addition there are works that try to automatize concrete parts of the diagnosis, such as dermoscopy analysis, using artificial intelligence [15, 16].

After the study of melanoma cancer problem we found that we are not just interested in a correct data analysis but we seek, above all, reliability. So our challenges properly represent knowledge, analyze each type of information, and establish collaborations between experts and tests. Thus we can not directly use any of the methods used until now, so we need a proposal specific to the problem that merges data mining techniques with the medical protocol. Attending to all these considerations DERMA is an interesting proposal for medical experts due to its ad hoc adaptation to the problem.

## 3. DERMA: Melanoma Diagnosis Based on Collaborative Multilabel Analog Reasoning

**Figure 1:**
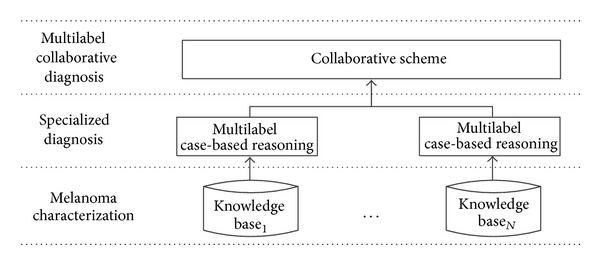
Melanoma diagnosis architecture based on collaborative multilabel reasoning.

DERMA is a platform that aids medical experts in melanoma diagnosis.  Figure 1 describes the DERMA architecture which addresses four different challenges identified during the collaboration with HCPB that range from data acquisition to diagnosis. The first challenge focuses on the creation of a melanoma ontology [17] based on a characterization of the domain performed through interviews with melanoma experts and the study of melanoma patterns. The second one is to create specialized subsystems in order to work with the different data sources. CBR is selected due to its suitability for working in environments where self-explanations are required. This step also considers how to organize the knowledge bases in a proper manner in order to improve its performance through distance metric learning [6]. The third challenge is to define a collaborative scheme [18] between the independent CBR subsystems based on the way by which experts work and which also includes mechanisms to manage exceptional situations. Finally, the last challenge is related to the complex task of making classifications of nontrivial patterns. In this sense, DERMA may be able to learn and diagnose better if richer patterns could be represented as a set of simple patterns. For this reason, we decide to extend the single-label CBR subsystems to multilabel [5] CBR subsystems. The next subsections describe these challenges in detail.

### 3.1. Challenge 1: Melanoma Characterization

The first step in the development of any knowledge-based system is to identify, understand, and gather data associated with the problem. These steps are nontrivial when the system is related to the health sciences domain because these data are usually characterized as being heterogenous and coming from several medical profiles involved in the prognosis. Moreover, experts often label data according to their interests and background, so attributes with different names may have the same meaning. Thus, the characterization and understanding of the relationships between all the data sources for planning the gathering of knowledge is a nontrivial task that requires time and consensus between experts.

**Figure 2:**
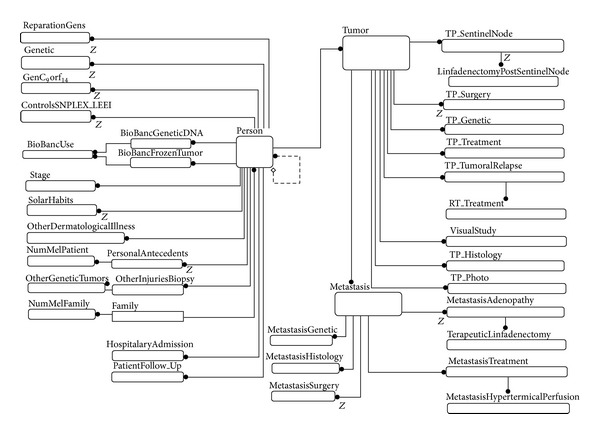
The melanoma relational model permits the definition of how to integrate data gathered from the different medical profiles. The model considers patient data, their family, generic information, tumors, metastasis, controls, and studies.

The concrete domain characterization was performed using data from more than three thousand patients with melanoma and contained reports from dermatologists, oncologists, surgeons, pathologists, and other specialists working in HCPB. As in the great majority of medical problems, data was heterogenous and distributed in different plain databases and many attributes were represented and stored differently according to the expert. For this reason, an ontology [17] with more than forty concepts was defined using the experts' point of view and data from international studies that examine specific aspects of the domain [19] divided in five groups: (1) person and family, (2) generic medical information, (3) tumors, (4) metastasis, and (5) controls and studies. Although this unified point of view permitted the integration between all data sources as Figure 2 shows through a relation model, there were pieces of information that could not be integrated and used in platform tests because experts did not have records regarding all patients. This is the reason why we decided to focus our work on the usage of nevus images analysis due to its availability and being outstanding between other data. More specifically, dermoscopy and confocal images were selected because they are two of the most promising techniques of image analysis for the diagnosis of melanoma. Dermoscopy is based on a microscopic image created by epiluminiscence microscopy (x10.30) and confocal reflectance is generated by the reflection of a coherent laser (x100) resolution at the level of the cell [3, 4].

In order to test if it was possible to identify equivalent patterns using the same data used by medical experts, we applied several data mining techniques. Melanoma diagnosis is based mainly on the ABCD rule which considers the following characteristics that are typically observed in this type of tumor: (A) a diameter greater than 5 mm, (B) color variation, (C) asymmetry, and (D) jagged edges. We tested *K*-means [20] and SOM [21] for extracting patterns due to our previous experiences in breast cancer diagnosis using these techniques [10]. *K*-means algorithm makes a partition of the domain in *K* clusters and SOM translates complex and nonlinear statistical relations contained in high-dimensional data into simple geometric relations on a low-dimensional space which provide an optimal organization. The results provided equivalent patterns to the ones identified by ABCD rules [13, 22] attending to medical criteria.

### 3.2. Challenge 2: Specific Diagnosis Using the Most Useful Knowledge

CBR systems solve new problems through an analogical procedure based on experiences represented by a set of cases stored in a case memory. Thus, CBR is able to justify the obtained solutions using analogies with previous problems which is crucial for experts. The way in which CBR works can be summarized in the following steps: (1) it retrieves the most similar cases from the case memory with a similarity function; (2) it adapts them to propose a new solution; (3) it checks if this solution is valid; and finally (4) it retains the useful information of the prognostic if it is necessary. All CBR steps turn around the case memory and its organization and how cases are retrieved determine its performance in terms of accuracy, specificity, sensitivity, and computational time [23].

There are two main possible memory organizations: flat and structured. A flat organization is the simplest way because cases are stored sequentially in a list. In such situations, the strategy to classify a new problem is to sequentially compare it with all the cases in that list using some similarity measure. The main shortcomings of this approach are that the more cases the case base contains, the higher the time of retrieval is and that the lack of organization may imply that useful cases are skipped. Structured memory organization focuses on improving both issues and many authors have tackled this issue from many points of view, such as representing the attributes in tree structures [24] or graphs [25], grouping cases by their similarity [26], and applying knowledge-intensive approaches [27] or data-intensive approaches [28]. Independent of the case memory organization, a distance function needs to be defined for comparing the similarity of cases. The ideal similarity function definition is not a trivial task because it depends on the domain and how data is related. There are even works that try to discover this similarity function using algorithms based on genetic algorithms [29]; the application of standard similarity functions such as Euclidean distance is the most frequent solution due to the complexity of identifying reliable distance metrics.

Owing to the importance of better determination of positive and negative melanoma cases we propose taking case memory organization and similarity function definition through a data organization based on distance metric learning (DML) [6]. DML is a technique used to identify a suitable distance metric based on the data projection that can be divided into four families [30]. The first two families are based on the supervision of the method: supervised and unsupervised DML. The last two families are based on a more concrete classification: based on support vector machines or kernel methods. In our case and in response to the characteristics of the problem, we are working with the supervised family. With this method we learn a metric that keeps all the data points from the same class close together and, at the same time, separates as far as possible the data points from different classes. We have learned a global distance metric that minimizes the distance between pairs of data included in the equivalence constraints and data pairs from the inequivalence constraints. With this process we obtained a case memory organized in a way that enables a better retrieval because positive and negative cases become distanced [31].

### 3.3. Challenge 3: A Global Diagnosis Using Independent and Specific Diagnosis

**Figure 3:**
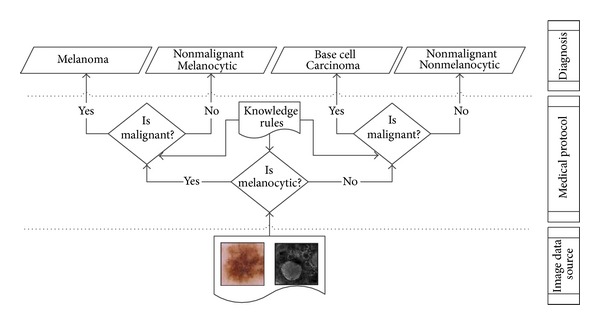
Medical diagnosis protocol schema followed by dermatological cancer experts.

The way in which melanoma is diagnosed takes into account different data sources and this makes it easy to apply a collaborative approach to classify new patients.  Figure 3 describes the medical process that experts consider, that is, effectively a collaborative process that determines if the new case is melanoma, basal cell carcinoma (BCC), or a nonmalignant tumor (melanocytic or not) according to the partial diagnosis using the confocal and dermoscopy images of the new patient.

The combination of approaches can be summarized [32] in (1) bagging, (2) boosting, and (3) stacking. Bagging and Boosting are based on the combination of the outputs using votes. In concrete bagging replicates N systems of the same approach but uses different data sources. In opposition boosting follows the same idea but defines models in order to complement them. Finally stacking [11] is based on heuristics that combine the outputs of several approaches. The most common voting methods [33] are (1) plurality, (2) contra-plurality, (3) borda-count, and (4) plurality with delete. All of them are based on the number of votes of a class (plurality) but with differences in the addition of plurality and decision of better class.

**Figure 4:**
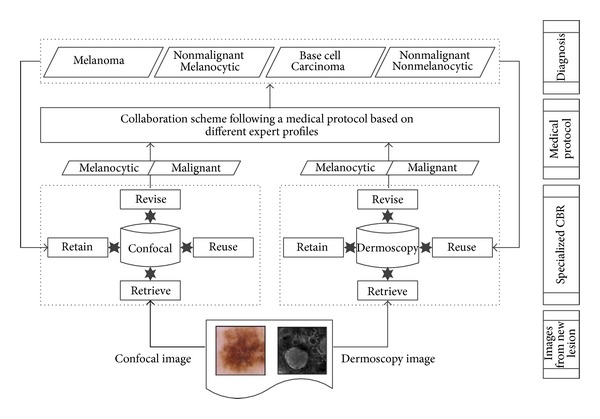
DERMA is based on a collaborative scheme between specialized CBR subsystems for melanoma cancer diagnosis following the medical diagnosis protocol.

There are several works that use collaborative systems that permit an improvement in well-known algorithms such as clustering using collaboration [34], to allow the classification using data of different complexity [35], or with different types of medical information [12]. There are so many general collaborative systems [36], but in melanoma classification we must consider specific characteristics that need the use of medical knowledge. The collaboration protocol should follow the one used by experts in melanoma that is to combine different decisions from different systems to build a more reliable solution using the individual ones, as it has been done in other problems [37]. In our case we are working with two different points of view (confocal and dermoscopic) from which we select the best classification from one of the systems depending on different criteria [38]. DERMA functional schema is shown in Figure 4 where we define two specialized CBR modules that follow the medical protocol for classification and later we combine the obtained results through collaborative criteria. Thus, the concrete characteristics of the domain [19] make it necessary to employ a different method from the general one. As we are using different attributes of the same data in each system, then the independence of the data is guaranteed, in contrast to the standard bagging. Analyzing the classification attributes, the voting method should be based on plurality, albeit with some specific conditions requested by medical researchers, who place more importance to the information from confocal microscopy because they consider it to be more reliable.

On the other hand, bearing in mind that the main goals are the improvement of the classification and minimizing the false negative situations, a knowledge rules module is introduced to ensure reliability in exceptional situations due to data oddities in a second layer of the collaborative system [39]. This module preprocesses the input data and creates a set of rules to help the whole classifier. It has been done using clustering in order to discover new patterns on the medical domain [22] and to detect particular behaviors on the data. Despite using a similar idea of [40], we preprocess the data in a nonbased interval way, where concrete values are detected and encapsulated in a rule. Moreover, our rules do not depend on each other and attributes are analyzed independently. The idea is to weight the single classification of each subsystem according to the reliability of the retrieved cases. The reliability of a case is based on a set of rules previously extracted from data. This new step adds a fine tuning to the classifier collaboration that leads to an improvement of the final classification.

### 3.4. Challenge 4: Multilabel Diagnosis

During the integration of the medical protocol an interesting aspect shows up: the final diagnosis is obtained from considering different medical profiles and/or classification patterns. This is the principle of multilabel classification problems where there is not a single class, but rather elements that are carved in parts to avoid information loss. We consider that the problem is better represented as two nondisjunctive classes such as melanocytic and malignant ones instead of using just a single class.

In the last few years we have witnessed an increase in the use of multilabel systems due to their better fitting to real problems and their ability to avoid information loss. Existing works to date have been divided into two distinct families. The first option is to adapt the dataset to work with single label algorithms instead of designing new algorithms. This group of techniques are known as problem transformation methods (PTM) and the main problem is that the unification of the different labels in a unique label is the loss of information. The most competent works in PTM for multilabel classification are MlKnn and RAkEL [41, 42] and both are recognized by the community as reference algorithms. MlKnn is a theoretical approach to multilabel classification which adapts the *k* recovered cases from the classical *k* nearest neighbor algorithm (Knn) [43] to multiple label problems. RAkEL is an ensemble platform that permits the classification of multilabel datasets by dealing with each label separately and combining the single-label results. It is publicly available through WEKA [44]. There are other interesting works in this field used by the community such as [45] where the authors present a pruned transformation that combines key points of several previous approaches and [46] that uses neural networks for multilabel classification. The second family addresses the problem with a modification of the classical algorithms to work purely multilabel and is known as algorithm adaptation methods (AAM). The most influential works in AAM include a boosting algorithm for text categorization [47], an adaptation of C4.5 [48] to deal with multilabel biological data [49], and a system that combines ranking methods with a predictor of the sets size [50].

Because we do not want to lose any information during the classification process, we focused on AAM approach. More specifically, we extended the phases of all CBR subsystems for working in multilabel mode. The four stages are designed as follows: (1) retrieval step follows the same pattern as the regular CBR algorithm. The system chooses the most similar cases according to the value obtained from a similarity function. The cases of the case memory that are more similar to the new case are the retrieved ones; (2) reusal stage has been adapted to multilabel classification in a probabilistic manner. It is based on the idea of counting the occurrences of each label and considers it positive if more than a half of the *k* retrieved cases have this positive label. This is similar to the idea proposed by multilabel *k*-nearest neighbor algorithm. A second step on the reuse phase considers the experience of each retrieved case (obtained through the retain feedback) weighting the recovered cases in an appropriate manner; (3) retaining phase to keep information on successes of the retrieved cases. This is the information by which we will weight the retrieved cases considering the successes of previous classifications; (4) revision, as in single-label, is performed by a medical expert.

## 4. Experiments and Results

The active collaboration with medical experts from HCPB has played an important role in all the steps taken to design DERMA and also in the preparation and study of experiments. Challenge 1 allowed us to characterize the domain taking into account the different medical profiles in order to define the ideal structure of the dataset. We are currently working with the Catalan Network of Melanoma in order to complete this dataset. Given the data available in melanoma database we have done two kinds of experiments: the first block covers the usage of dermoscopy and confocal data for testing the collaborative approach of the CBR subsystems in both data, and the second one works with multilabel melanoma data in order to test the complete DERMA.

The next points summarize the data used for experimentation and the most important milestones achieved and the outstanding results following the cited two blocks of experiments.

### 4.1. Testbed

The most used techniques to gather information from tissue are the dermoscopic and the confocal analysis. Confocal microscope is the most precise and the one that medical experts consider as world class. Nevertheless, a negative point is that the confocal analysis is a long and expensive test, so the number of available cases is limited. Due to this situation, the data set used in this work is composed of 150 cases of suspicious lesions. For all these cases we have information related to confocal and dermatoscopic images and the histology that corroborated diagnosis. Attending to the considerations of the medical experts that have created this set, it includes enough cases from each kind of illness to be representative of the domain. Then, in medical terms it is an appropriated case memory for this study. Detailing the instances, dermoscopy information has forty-one fields and confocal microscopy, due to its higher resolution, contributes to data from eighty-three different attributes. This data has been configured in two different manners: the first as a single-label set of dataset where each classification process uses the appropriate attributes to classify one class and the second exploits all the properties of the data to classify all the possible classes at a time.

### 4.2. Experimental Framework and Configurations

Experimentation has been carried out according to the medical purposes in order to analyze the most interesting results for experts, such as false positives. The experimentation with medical data from confocal and dermoscopy images has tested DERMA using different configurations and analyzing sensitivity, specificity, and accuracy. The study considers two independent CBR subsystems with a basic decision combination, with the use of rules obtained through the use of preprocessing algorithms and with the application of DML to the original data. In addition, we tested the accuracy of the two independent CBR systems (one for confocal data and another for dermatoscopy). All the CBR systems used in experimentation are configured with one-nearest neighbor algorithm with normalized Euclidean distance as retrieve function and classify a single class at a time. In the case of the plain combination platform, the medical consensus is to use 0.5 as confocal threshold and double of the distance between the new case and the best confocal case as dermatological one. And the other stages use the threshold weighted by the preprocessed rules. This experiment framework has been tested applying a leave one out to the original data to obtain the average accuracy of those systems. The final challenge was focused on the use of multilabel data and, as a consequence, the use of multilabel CBR subsystems in our collaborative platform. For this experimentation we considered two classes in each instance (melanocytic and malignant) that offer experts the whole range of classifications of a nevus as in the single-label case. In addition we have performed a *t*-test with 95% confidence level between each configuration of DERMA and the previous one to establish the results of significance.

### 4.3. Results Using Single-Label Data

Having described the experimental framework we were able to analyze the results obtained.  Table 1 shows sensitivity, specificity, and accuracy rates classifying new injuries using the two independent CBR and the three different collaboration schemes defined in DERMA: with rules, without them, and with the DML module. The results on Table 1 show the four possible classes that are considered by the medical protocol. The statistical significance of the results is represented with an ↑ if it is significantly better and a (—) if it is equivalent. The results obtained highlight that (1) the different layers added to DERMA achieve better classification results than the plain combination DERMA or the independent noncollaborative systems; (2) the use of the combination of both types of images with the help of preprocessing obtained rules leads to an important increase in sensitivity and specificity rates, the most important results for medical experts; (3) using the DML technique in order to better classify the new cases, we accomplish the desire of medical experts that is to avoid false negatives allowing the successful diagnosis of all patients; (4) it is easier to classify nonmalignant cases, it seems to be related to the fact that we use a real world dataset that fits the characteristics of the population and there are more patients with nonmalignant cases than with malignant ones; (5) the nonmelanocytic cases are better classified due to the characteristics of the problem; (6) the *t*-test results show that any improvement of DERMA has a significantly negative effect.

### 4.4. Results Using Multilabel Data

The results obtained by DERMA are shown in Table 2 and show the percentage of successes considering the four possible nevus classifications. This table is formed by the sensitivity, specificity, and accuracy results obtained using a noncollaborative protocol with confocal and dermoscopy data, respectively, and with a collaborative pattern with the combination of both data using plain combination, preprocess rules, and distance metric learning. As in single label the significance is represented with an ↑ if the result is significantly better and a (—) if it is equivalent. The results obtained highlight that (1) as in single-label classification the different layers added to DERMA achieve better classification results than the plain combination DERMA or the independent noncollaborative systems; (2) the results using the whole system permit the successful diagnosis of all cases. Although we achieve hundred percent accuracy we are not facing a foolproof system but one which knows how to work with the peculiarities of the domain, just as medical experts; (3) the classification of all classes in an unique step does not lose any kind of information; (4) each enhancement of DERMA is positive with statistical significance or, at least, equivalent in comparison to the nonuse of the improvement.

In addition to this experimentation and in order to test the strength of the method we made a previous study where we tested the multilabel classification part of DERMA with other kinds of data (due to the absence of more melanoma multilabel datasets) and in comparison with other multilabel classification platforms [51]. This work allowed us to tune the characteristics of our platform and to validate it as a competent method in this kind of classification. As general purpose repositories such as UCI [52] do not give enough multilabel datasets, we tested DERMA with seven synthetic datasets and the three most common real world multilabel datasets [5]. The obtained results show that DERMA results are equivalent to the ones obtained by reference platforms RAkEL and MlKnn. We would like to highlight that the multilabel classification reduces the steps required to obtain the same result, thus reducing the computational costs.

### 4.5. Global Results and Discussion

Once the results of our platform have been presented in terms of performance, we will discuss the system: its characteristics and its applications. As we have seen DERMA is an on-demand application that fits the needs of medical experts in melanoma diagnosis. Attending to the analysis of possible helpful and harmful issues from internal and external origins, we could point that DERMA has its strengths in the fact that it improves the sensitivity and specificity rates, which is important for experts, and gives classification explanations. These explanations are based on the retrieved cases that provide the medical experts with an explanation of the similarity between the new case and the prediction. Moreover it is based on an increasingly common cancer that, attending to the American Academy of Dermatology, improves the recovery results with a proper early diagnosis offering an important opportunity to research. On the other hand, we must deal with external threats such as data availability, the medial protocol that is dynamic, and the changes in data from new studies. All theses weaknesses are being covered through a data platform and with the scalability and fine tuning of DERMA that allows a wide range of changes.

## 5. Conclusions and Further Work

Melanoma cancer is a growing problem in our society due to the increasing number of cases. The characteristics of this disease and the different types of techniques and professionals involved in the diagnosis make it important to design a platform that covers the entire problem. DERMA was born to achieve this objective using all the features extracted from the preliminary analysis of the problem. The platform is a decision support system to help medical experts in their diagnosis. The general architecture permits the integration of several data sources that correspond to the different medical profiles and techniques involved in a melanoma diagnosis. Each data source is used to configure a single CBR subsystem that performs a single classification. The combination of all CBR subsystems through a medical protocol scheme results in a final collaborative diagnosis.

Every challenge we have addressed has improved the results of previous steps. We started our work with a single classification, using a single CBR system with just one kind of data. This first step allows us to determine the base accuracy. Later, we propose a collaborative diagnosis following the medical protocol, where different subsystems make a unified prognostic with different data sources. This module performs the same process used by medical experts combining different criteria. The basic collaboration was followed with the application of enhancements on the knowledge base organization and the collaborative process. This work tunes the system in order to improve the sensitivity and specificity rates. Finally, we extended the work to the use of multilabel data due to the domain characteristics. This last step offers good results and keeps the door open to richer datasets. We could summarize that the results obtained by DERMA during the test process were the ones expected by medical experts. Nowadays the most outstanding problem remains to be the lack of enough melanoma data, but we are developing a data managing application that will solve this problem. Once we obtain the complete data that fits all the melanoma features, we will recheck our results in order to use DERMA system to help in the day-to-day assistance which is our main future goal.

Moreover, analog reasoning and hybrid systems, such as collaborative and multilabel CBR, are hot research topics particularly in the area of health and medicine. It is crucial to have a framework as flexible as the one offered by this family of techniques. They are highly reliable within the community of data mining. Some of these processes are being explored in new fields such as social networks and marketing areas. Techniques such as analog reasoning exploit the high capacity of the computer to find patterns that lead to new and useful information for the user. Our proposals could be moved to these new areas in order to take care of new problems. This is the second part of our further work.

## Figures and Tables

**Table 1:** Sensitivity, specificity, and accuracy results obtained in melanocytic, melanoma, and BCC classification through the different DERMA challenges: the noncollaborative CBR classification which only uses dermoscopy data, the noncollaborative CBR classification which only uses confocal data, the plain collaborative system, the collaborative system that enhances the collaboration with preprocessing rules, and the collaborative system with a DML organized case memory. Each result shows the *t*-test comparison between the result obtained on this DERMA configuration in comparison with the previous one using 95% of confidence level. This is presented with an (↑) if it is significantly better and (—) if there is no significant difference.

	Nonmalignant		Malignant	
	Melanocytic	Nonmelanocytic	Melanocytic	Nonmelanocytic
			(melanoma)	(BCC)
Sensitivity results				
Dermoscopy CBR	75%	80%	73%	81%
Confocal CBR	74% (—)	92% (↑)	73% (—)	92% (↑)
Collaborative	80% (↑)	94% (—)	70% (—)	92% (—)
Collaborative + rules	95% (↑)	95% (—)	81% (↑)	92% (—)
Collaborative + rules + DML	100% (↑)	100% (↑)	100% (↑)	100% (↑)

Specificity results				
Dermoscopy CBR	95%	99%	92%	96%
Confocal CBR	99% (↑)	98% (—)	96% (↑)	95% (—)
Collaborative	96% (—)	97% (—)	95% (—)	96% (—)
Collaborative + rules	99% (—)	99% (—)	98% (↑)	100% (↑)
Collaborative + rules + DML	100% (—)	100% (—)	100% (↑)	100% (—)

Accuracy results				
Dermoscopy CBR	90%	96%	87%	96%
Confocal CBR	88% (—)	95% (—)	90% (↑)	95% (—)
Collaborative	92% (↑)	94% (—)	89% (—)	95% (—)
Collaborative + rules	98% (↑)	99% (↑)	94% (↑)	99% (↑)
Collaborative + rules + DML	100% (↑)	100% (↑)	100% (↑)	100% (↑)

**Table 2:** Sensitivity, specificity, and accuracy results obtained in multilabel classification using dermoscopy data, confocal data, and both types of data with a collaborative system and using the different DERMA modules. Each result shows the *t*-test comparison between the result obtained on this DERMA configuration in comparison with the previous one using 95% of confidence level. This is presented with an (↑) if it is significantly better and (—) if there is no significant difference.

	Sensitivity	Specificity	Accuracy
Multilabel dermoscopy	86%	89%	92%
Multilabel confocal	93% (↑)	97% (↑)	96% (↑)
Multilabel collaborative	91% (—)	96% (—)	95% (—)
Multilabel collaborative + rules	94% (↑)	99% (↑)	98% (↑)
Multilabel collaborative + rules + DML	100% (↑)	100% (—)	100% (↑)
